# Carbohydrate Quality Is Independently Associated with Cardiometabolic Risk in Chinese Individuals with Impaired Glucose Tolerance

**DOI:** 10.3390/nu17071123

**Published:** 2025-03-24

**Authors:** Natural H. S. Chu, Yelia Yu, Jie He, Cynthia R. H. Li, Seong I. Pai, Kathy H. T. Leung, Ronald C. W. Ma, Juliana C. N. Chan, Elaine Chow

**Affiliations:** 1 Department of Medicine & Therapeutics, The Chinese University of Hong Kong, Prince of Wales Hospital, Hong Kong SAR, China; 2 UNC Gillings School of Global Public Health, The University of North Carolina at Chapel Hill, Chapel Hill, NC 27517, USA; 3 Department of Physiology, University of Toronto, Toronto, ON M5S 1A1, Canada; 4 School of Medicine, Dentistry and Nursing, University of Glasgow, Glasgow Q12 8QQ, UK; 5 Li Ka Shing Institute of Health Sciences, The Chinese University of Hong Kong, Prince of Wales Hospital, Hong Kong SAR, China; 6 Hong Kong Institute of Diabetes and Obesity, The Chinese University of Hong Kong, Prince of Wales Hospital, Hong Kong SAR, China

**Keywords:** fibre, carbohydrate quality, prediabetes, fibre/CHO ratio, cardiometabolic syndrome

## Abstract

**Background/Objectives**: Dietary manipulation with carbohydrate restriction has been extensively investigated in diabetes prevention programmes. Carbohydrate (CHO) quality, rather than quantity, is associated with various metabolic outcomes. Few studies examined the fibre/CHO ratio on lipid profiles, liver fat and insulin resistance in individuals with impaired glucose tolerance (IGT). **Methods:** In this comprehensive cross-sectional study, we evaluated the association of carbohydrate-related nutritional factors with metabolic parameters in a cohort of 177 Hong Kong Chinese with impaired glucose tolerance (IGT). The subjects underwent a 75 g oral glucose tolerance test (OGTT) with measurement of plasma C-peptide and lipid profiles, body composition, transient elastography, and three-day food records. The fibre/CHO ratio is calculated by dividing fibre intake by total carbohydrate intake (in grams). **Results:** The median (IQR) age of the study cohort was 60 (54–62) with a mean ± SD BMI of 26.7 ± 3.9 kg/m^2^, and 40.7% were female. A higher carbohydrate quality, measured as fibre/CHO ratio, was inversely correlated with triglycerides (r = −0.305, *p* < 0.001) and positively correlated with High-density lipoproteins cholesterol (HDL-C) (r = 0.354, *p* < 0.001). These associations remained significant after adjusting for age, gender, lipid-lowering drugs, total calorie, macronutrient and sugar intake, physical activity and sodium/potassium ratio. Blood pressure, liver fat and insulin resistance were also associated with the fibre/CHO ratio after the adjustment of these confounding factors. Consuming more than 5.5 g of fibre per 100 g carbohydrate was associated with lower serum triglycerides. **Conclusions**: Our results highlight the potential for using the fibre/CHO ratio as a metric for daily carbohydrate quality and the importance of addressing both carbohydrate quality and quantity in designing dietary interventions to reduce cardiometabolic risk.

## 1. Introduction

Blood glucose levels are influenced by the amount of carbohydrates consumed. Diets high in carbohydrates (CHO) have been linked to increased hyperglycaemia and suppressed islet functions, including insulin and C-peptide responses. Additionally, they affect plasma lipoproteins, including total cholesterol, high-density lipoproteins (HDLs) cholesterol, low-density lipoproteins (LDLs) cholesterol, and, particularly, triglyceride levels [1,2]. Higher carbohydrate intake increases basal and post-glucose energy expenditure and oxidation stress, leading to an increase in the production of circulating triglycerides and the risk of metabolic dysfunction-associated steatotic liver disease (MASLD) [3]. Therefore, restricting carbohydrate intake could attenuate liver fat, hyperglycaemia and hypertriglyceridaemia. However, there are potential adverse health effects associated with a low-carbohydrate diet, including an increased risk of mineral deficiency, lack of essential vitamins, and reduced intake of dietary fibres [4]. A meta-analysis and systematic literature review of 66 articles on 50 randomised controlled clinical trials (RCTs) showed that moderately low-carbohydrate or low-carbohydrate diets might be useful for weight loss, whereas a very low-carbohydrate/ketogenic diet is ineligible for patients with diabetes [5], with a potential risk of abnormal lipid profiles [6]. These untoward effects were not a persistent consequence in reducing liver fat [7,8] and may be alleviated by consuming high-quality carbohydrates [9].

In this light, several studies have suggested that dietary manipulation in carbohydrate and fibre contents can help with weight loss and reduce plasma glucose, HbA1c, liver fat and lipoproteins, potentially preventing high-risk individuals and those with type 2 diabetes [2,8,10]. A diet high in carbohydrates and fibre, mainly from plant-based sources such as legumes, vegetables, fruits, and whole cereals, rather than simple sugars from beverages, may be beneficial for treating diabetic patients and reducing postprandial lipid abnormalities [11].In prospective cohort studies, high-carbohydrate diets, particularly those emphasising foods with high-quality carbohydrates, low glycaemic index and load, and high fibre content, have been associated with decreased intermediate cardiometabolic risk factors [12]. In line with a meta-analysis of 23 randomised control trials examining the effect of dietary fibre on lipid profiles, increased fibre intake led to lower total cholesterol and LDL by the mean of 0.23 mmol/L and 0.14 mmol/L, but not in triglycerides and HDL [13]. Notably, soluble fibre supplementation has been clinically shown to lower cholesterol for cardiovascular health due to an increase in viscosity in the small bowel that reduces cholesterol absorption [14]. In a meta-analysis of 181 randomised clinical trials to investigate the effects of soluble fibre supplementation on blood lipid parameters in adults, each 5 g/d increment in soluble fibre supplementation reduced total cholesterol, LDL and triglycerides by 0.158, 0.144 and 0.041 mmol/L, respectively [15]. A meta-analysis of studies on nutraceutical-drug interactions showed that combining psyllium fibre supplementation with statin therapy achieved a greater reduction in LDL compared to using a statin alone [16].

In addition, high-protein diets have significantly reduced triglycerides in patients with type 2 diabetes compared to low-protein diets [17]. The ratio of energy from fat and carbohydrates in the diet can also affect the impact of a high-protein diet on weight and triglycerides [18]. Substituting dietary carbohydrates for protein and fat seems to have a beneficial effect on several cardiovascular risk markers in patients with diabetes [19]. However, in a long-term prospective community-based cohort, a high dietary protein/carbohydrate ratio has been associated with an increased risk of metabolic syndrome in men [20]. In a separate study examining the impact of a high carbohydrate diet versus a high monounsaturated fatty-acid diet on patients with insulin-dependent diabetes, no significant variances in glycaemic parameters were noted with the high monounsaturated fatty-acid diet [21]. Much of the controversy between advocates and detractors of other dietary macronutrients or fibre in carbohydrate intake.

Meanwhile, the presence of essential minerals like potassium in carbohydrates may positively impact triglycerides [22]. For instance, consuming a variety of whole grains, vegetables, and fruits can increase dietary fibre, which may also affect the sodium/potassium ratio. Lower sodium intake and higher potassium bioavailability may influence intracellular fluid and cell function [22], potentially helping to manage cardiometabolic syndrome.

Our aim is to understand how dietary factors related to carbohydrates can affect metabolic health in people with impaired glucose tolerance (IGT). We hypothesised that a higher fibre/CHO ratio will be associated with lower cardiometabolic risk, including lower insulin resistance, lipids and triglycerides. Through this study, we hope to create a straightforward nutritional index that can assess the quality of carbohydrates. Ultimately, the findings could help people with IGT make better dietary choices to improve their metabolic health and reduce the risk of cardiovascular disease.

## 2. Materials and Methods

### 2.1. Subjects

This is a prospective, cross-sectional, observational study in Chinese prediabetes. We accessed that baseline data on subjects who were screened to identify individuals with IGT for a 12-month randomised clinical trial, which evaluates the effects of a technology-assisted diabetes prevention programme for the prevention of glycaemic deterioration. Written informed consent was obtained from eligible subjects conducted at the Prince of Wales Hospital (PWH) of the Chinese University of Hong Kong. Subjects were recruited from patients attending the PWH medical outpatient clinics or through self-referrals via advertisements.

### 2.2. Inclusion and Exclusion Criteria

In this study, we included subjects aged between 18 and 65 years, with a body mass index (BMI) ranging from 18 to 40 kg/m^2^. Eligibility required subjects to be non-pregnant and not currently lactating, to have no history of diabetes, and to not be undergoing treatment with anti-diabetic or anti-obesity medications, have known uncontrolled thyrotoxicosis, current use of steroids, recent or current alcohol or drug abuse. Individuals who have participated in a weight loss programme within three months prior to screening were excluded from the study. After an overnight fast of at least 8 h, all subjects underwent a 75 g oral glucose tolerance test (OGTT). Glycaemic status was classified according to the American Diabetes Association (ADA) criteria: impaired glucose tolerance (IGT) was defined as having a 2 h plasma glucose level between 7.8 mmol/L and 11.0 mmol/L [23].

### 2.3. Anthropometrics and Body Composition

Body weight and percentage of body fat were assessed using the Tanita bioelectrical impedance analysis system (Model: TBF-410 Body Composition Analyzer, Tanita Corporation, Tokyo, Japan) [24,25], while they were wearing light clothing and no shoes. Height was measured using a stadiometer to the nearest 0.1 cm for the calculation of Body Mass Index (BMI). The waist measurement was taken at the level of the umbilicus, and the hip measurement was taken around the most prominent part of the buttocks, just below the iliac crest [26].

### 2.4. Biochemical Profiles

Blood samples were collected through a venous catheter from an antecubital vein into vacutainer tubes containing fluoride and EDTA (ethylenediaminetetraacetic acid) at 6 time points: fasting, 15, 30, 60, 90, and 120 min for measurements of plasma glucose and plasma C-peptide (CP). The CP concentration was measured using radioimmunoassay (Novo Nordisk, Copenhagen, Denmark), with a lowest detection limit of 0.1 nmol/L. The intra-assay coefficient of variation (CV) was 3.4%, while the inter-assay CV was 9.6% [27]. Plasma glucose was assayed using the hexokinase method at a certified commercial laboratory using enzymatic methods in accordance with established standards [28]. Fasting serum lipids (total cholesterol, triglycerides, LDL-C, HDL-C, and non-HDL-C) were measured using the direct quantitation at a certified commercial laboratory. LDL-C was calculated using the Friedewald equation [29].

In our study, we calculated the steady state of insulin resistance (HOMA2-IR), insulin secretion (HOMA2-%B), and insulin sensitivity (%) using the Homeostasis Model Assessment (HOMA2) Calculator v2.2.3, which we downloaded from http://www.dtu.ox.ac.uk. [30], accessed on 23 June 2022. We analysed the HOMA2-IR score as a continuous value, where a high value indicates increased insulin resistance. Additionally, we calculated the area under the curve (AUC) of plasma glucose and C-peptide using the trapezoidal rule during the oral glucose tolerance test (OGTT) to cover all aspects of our study [31].

### 2.5. Hepatic Parameters Measurements:

Transient elastography (FibroScan, Echosens, Paris, France) was conducted by experienced operators to obtain the controlled attenuation parameter (CAP) and liver stiffness measurement (LSM) using either an M probe or an XL probe which is suitable for subjects. Transient elastography was conducted following the OGTT after a fasting period of at least two hours to the examination. Liver stiffness results were measured in kilopascals (kPa). The examination was deemed reliable if at least 10 valid acquisitions were obtained, with an interquartile range over a median of ≤30% for LSM [32]. Hepatic steatosis and severe hepatic steatosis were defined as CAP ≥ 248 dB/m and ≥280 dB/m, respectively [33,34]. Baseline LSM  ≥  10 kPa was considered a suggestive of advanced chronic liver disease in decompensation.

### 2.6. Dietary Evaluation and Physical Activity

Subjects who were eligible and gave written consent were asked to keep a record of their regular dietary intake using three days food records before randomization. The three-day food records have been shown to be accurate in capturing macronutrients when compared with Food Frequency Questionnaires (FFQ) in a local study in people with IGT and when compared with image-based smart phone app [35,36]. Each log (Supplementary Materials) covered two weekdays and one weekend day to accurately capture variations in food intake between those periods [37]. Subjects were required to provide details of the quantities of meals and beverages consumed and were trained to estimate serving sizes using standardised local bowls and plates by a research dietitian/nutritionist. If subjects dine out or are unsure about portion sizes, they can refer to the local government website, which offers detailed estimations for various individual foods and cuisines, adhering to standard portion sizes (photo booklet) published by the Centre for Food Safety, Hong Kong [38]. After returning the food records, our research dietitian carefully reviewed the records to completeness and accurateness, with confirmation from another research nutritionist. The food records were then analyzed using nutritional analysis software (eSHA Food Analysis and Labelling Software version 4.0) for energy, macronutrients, and total dietary fibre content. Dietary carbohydrate ratios were calculated by dividing protein, fat, or fibre by carbohydrates, while the dietary sodium/potassium ratio was calculated by dividing dietary sodium intake by dietary potassium intake (all dietary intakes were the average of three days’ food records). Physical activity levels were recorded using the International Physical Activity Questionnaires (IPAQ) in its Chinese version [39].

### 2.7. Statistical Analysis

For comparisons, we conducted a comprehensive statistical analysis employing a variety of tests, including Mann–Whitney U test, Student’s *t*-test, chi square (χ^2^), Fisher’s exact test, or analysis of variance (ANOVA), as appropriate. Descriptive statistics included the mean (standard deviation [SD]) for normally distributed data and the median (interquartile range [IQR]) for non-normally distributed data, as appropriate.

In this cohort of 177 subjects with prediabetes, we utilised Spearman correlation analysis to examine the relationships between carbohydrate-related ratios and lipid profiles, blood pressure, liver fats, and insulin response. In addition to age and gender, we adjusted for use of lipid-lowering drugs and antihypertensive, which will influence systolic blood pressure and lipid profiles. Total physical activity, total calories and macronutrient intake were adjusted as potential confounders for the relationship between Fibre/CHO ratio and the dependent variables of interest. We constructed a multivariate linear model of the fibre/CHO ratio against systolic blood pressure, triglycerides or HOMA-IR with age, gender, lipid-lowering and antihypertensive drugs as covariates (base model). The base model was adjusted for age, gender, lipid-lowering drugs and antihypertensive drugs. In model 1, we further included total energy and daily consumption of other macronutrients intake on top of the base model. In model 2, we included physical activity on top of model 1. In model 3, we also included the dietary sodium-to-potassium ratio to examine the fibre/CHO ratio on top of model 2. In model 4, we adjusted for education status and alcohol consumption on top of model 2. Binary regression was used to investigate the correlation between the quartile of fibre/CHO ratio and raised serum triglycerides (>1.7 mmol/L), adjusting for age and gender in model 1, and lipid-lowering and antihypertensive drug use in model 2. All data were analysed using version 26.0 of the Statistical Package for Social Sciences (SPSS Inc., Chicago, IL, USA).

## 3. Results

A total of 502 subjects underwent the 75-g OGTT; 250 were subjects with normal postprandial 2 h glucose levels, 177 had IGT, and 64 had diabetes. A total of eight subjects withdrew consent, and three subjects were excluded by other eligibility criteria. One hundred seventy-seven subjects were recruited who fulfilled the American Diabetes Association (ADA) criteria for impaired glucose tolerance. We collected their anthropometric, biochemical parameters, 3-day dietary records, and physical activity at baseline. The median age of the study population was 60 (54–62) years and 59% were female. The baseline demographics of the study are shown in Table 1. Female IGT subjects had a lower percentage of lipid-lowering and antihypertensive drugs, weight, waist circumferences, diastolic blood pressure, fasting CP, and HOMA-IR, but higher body fat, total cholesterol, HDL, and insulin sensitivity compared to males (*p* < 0.05). They also had lower macronutrients and sodium intake but higher fibre/CHO ratio than males (Table 2). However, there were no significant differences in glucose response, liver stiffness, liver fat, fibre intake, and physical activity.

### 3.1. Correlations Between Nutritional Index, Lipid Profiles, and Liver Fat

**Figure 1:**
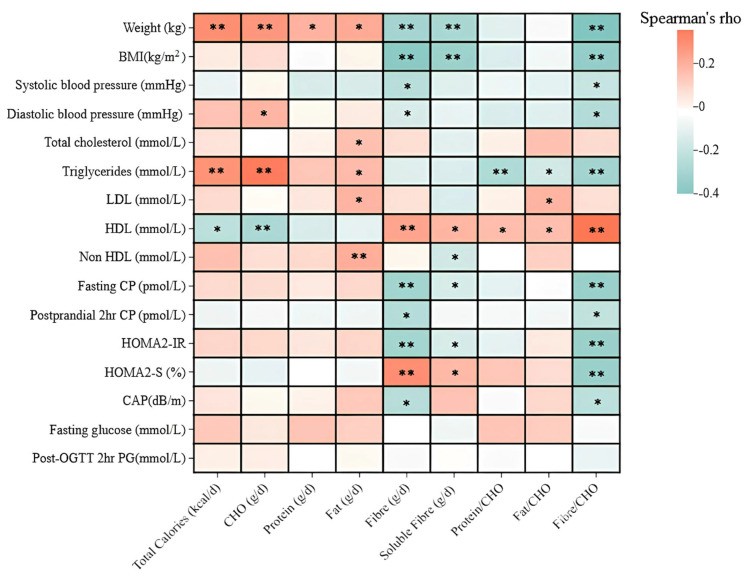
Spearman correlation in biochemical parameters and macronutrients, fibre and nutritional index. Abbreviation: BMI: body mass index, HDL: high-density lipoprotein cholesterol, LDL: low-density lipoprotein cholesterol, HOMA2-S: homeostatic model assessment for insulin sensitivity, CP: C-peptide, CHO: carbohydrates, OGTT: oral glucose tolerance test, HOMA2-IR: homeostatic model assessment 2 for insulin resistance. CAP: controlled attenuation parameter. ** *p* < 0.0001, * *p* < 0.05.

In the correlation analysis, higher fat intake (r = 0.181, *p* = 0.017) and fat/CHO ratio (*r* = 0.163, *p* = 0.032) were correlated with higher LDL levels. Lower energy intake and CHO intake were associated with lower plasma HDL levels, but higher fibre intake (r = 0.225, *p* = 0.003), protein/CHO ratio (r = 0.156, *p* = 0.038), fat/CHO ratio (*r* = 0.153, *p* = 0.042) and fibre/CHO ratio (*r* = 0.354, *p* < 0.001) were correlated with higher plasma HDL (Figure 1).

Furthermore, higher energy intake (*r* = 0.275, *p* < 0.001), CHO intake (r = 0.339, *p* < 0.001), and fat intake (r = 0.163, *p* = 0.030) were correlated with higher plasma triglyceride levels. However, no association was found between plasma triglyceride levels and intakes of protein and fibre. On the other hand, higher protein/CHO ratio (r = −0.267, *p* < 0.001), fat/CHO ratio (*r* = −0.163, *p* = 0.030), and fibre/CHO ratio (*r* = −0.305, *p* < 0.001) were correlated with lower plasma triglyceride levels. The controlled attenuation parameter (CAP) was inversely correlated with fibre (*r* = −0.241, *p* = 0.017) and fibre/CHO ratio (*r* = −0.225, *p* = 0.026), respectively (Figure 1).

### 3.2. Correlations Between Nutritional Index and Insulin Response

Our study found correlations between dietary intake and insulin response. Higher total calories and fat intake were positively correlated with higher fasting CP (*r* = 0.183, *p* = 0.038 and *r* = 0.189, *p* = 0.032) and HOMA2-IR (*r* = 0.202, *p* = 0.022 and *r* = 0.201, *p* = 0.022). Conversely, dietary fibre and fibre/CHO ratio showed a negative correlation with fasting CP (*r* = −0.281, *p* = 0.001 and *r* = −0.357, *p* < 0.001), 30 min CP (*r* = −0.223, *p* = 0.011 and *r* = −0.284, *p* = 0.001), 2 h CP (*r* = −0.253, *p* = 0.004 and *r* = −0.266, *p* = 0.002), and HOMA2-IR (*r* = −0.278, *p* = 0.001 and *r* = −0.367, *p* < 0.001). There were no significant correlations of other macronutrients and nutritional indices with insulin responses and glucose variables as noted in Figure 1.

In our multivariate analysis, we selected systolic blood pressure, triglycerides, CAP and HOMA2-IR as the key dependent variables to examine relationships with the fibre/CHO ratio. The base model revealed a significant association between these dependent variables and the fibre/CHO ratio even after adjusting for age, gender, lipid-lowering, and antihypertensive drugs (Table 3). Since lipid-lowering and anti-hypertensive drugs affect cellular metabolism, it is important to account for these confounding factors, as they can significantly influence cardiometabolic risk [40,41]. The associations persisted after further adjustments for macronutrients, sodium/potassium, physical activity, education status and alcohol consumption, demonstrating the robustness and reliability of our findings (Table 3). However, we did not observe the significant associations between protein/CHO or fat/CHO ratio and systolic blood pressure, triglycerides, liver fat, or insulin resistance after adjusting for age and gender (*p* > 0.05).

A binary logistic regression model was further conducted to investigate the relationship between quartile-categorised fibre/CHO ratio and hypertriglyceridaemia (>1.7 mmol/L). We showed that higher fibre/CHO ratios, such as those in the 3rd (5.5 g to 7.6 g of fibre per 100 g of carbohydrates) and 4th quartiles (>7.6 g of fibre per 100 g of carbohydrates), were associated with a lower risk of developing hypertriglyceridaemia. These findings remained significant even after adjusting for age and gender in Model 1 and lipid-lowering and antihypertensive drugs in Model 2 (Table 4). This underscores the profound impact of our findings on the quality of carbohydrates and cardiometabolic health.

## 4. Discussion

In this cross-sectional analysis, we observed that a higher fibre/CHO ratio was associated with lower cardiometabolic risk, including systolic blood pressure, triglycerides, liver fat and insulin resistance in subjects with IGT independent of age, gender, lipid-lowering and antihypertensive drugs, physical activity, other macronutrient intake, and sodium/potassium ratio. Moreover, a simple nutritional index fibre/CHO ratio, compared to other macronutrients to CHO ratio, shows a stronger association with triglycerides and HDL, than dietary fibre alone. We observed consistent findings with two cross-sectional studies involving individuals both with and without diabetes in Japan and Brazil, wherein the fibre/CHO ratio was associated with cardiometabolic parameters, including triglycerides and HDL [42,43]. Additionally, the quality of carbohydrates was linked to blood pressure control [44] and MASLD [45]. However, their studies did not adjust for other potential nutritional factors, such as minerals, which are may attenuate body fat and insulin resistance [46]. Our result shows that the associations of fibre/CHO ratio with cardiovascular risk remained robust after adjusting for the dietary sodium/potassium ratio [47,48]. To quantify adequate fibre intake, binary regression was conducted to investigate whether consuming more than 5.5 g of fibre per 100 g carbohydrate was associated with a lower risk of developing hypertriglyceridaemia independent of lipid-lowering drugs among those people at high risk of diabetes. Although the effect size of fibre/CHO and triglycerides itself was modest, it may be effective in modulating triglyceride levels when combined with other measures of control of alcohol, total sugar and fat intake and/or pharmacological agents clinically.

Chronically elevated blood glucose leads to insulin resistance in adipose tissue, increases intracellular triglyceride hydrolysis, and releases free fatty acids into the circulation and liver, favouring hypertriglyceridaemia and fatty liver and increasing the risk of cardiovascular diseases [49,50,51]. The quality of carbohydrates rather than quantity has the most potent effect on several non-communicable diseases, with a 15–30% decrease in all-cause and cardiovascular-related mortality and the incidence of coronary heart disease, stroke, and type 2 diabetes mellitus [52,53]. In a large longitudinal cohort of 3325 subjects with an average of 4.2 years of follow-up, changes in triglycerides/HDL were associated with peripheral insulin resistance and 2 h post-load insulin levels [54]. Therefore, we specifically investigated the role of the fibre/CHO ratio, a measure of the quality of dietary carbohydrates, in influencing cardiovascular risk and insulin resistance in subjects with IGT. This ratio was chosen because it provides a comprehensive view of carbohydrate quality, considering factors beyond the glycaemic index. Fibre/CHO ratio encapsulates not only the glycaemic load and effects on glycaemic excursions but also the broader impact of fibre on gut transit time, fermentation and changes in gut microbiota when combined with different quantities of carbohydrates. The glycaemic index is based on 50 g of carbohydrates. This measurement can vary in portion sizes, and the rating applies only to individual foods without considering the effects of mixed meals and cannot characterise contributions of multiple carbohydrates to diet quality [55].

### 4.1. Impact of Carbohydrate Quantity on Lipid Profiles and Liver Fat

Subjects with diabetes tend to eliminate carbohydrate intake because carbohydrates elevate blood glucose levels. In our results, we also observed that a higher intake of carbohydrates was positively correlated with body weight, diastolic blood pressure and plasma triglycerides, which inversely correlated with HDL levels (Table 3). However, in another retrospective study in patients with type 1 diabetes, those patients had a lower carbohydrate intake, less than 50% of their total caloric intake, correlated with a longer duration of diabetes [56]. In a multicentre, open-label, randomised controlled trial, 1678 people with impaired glucose regulation were randomised into lifestyle modification with or without metformin treatment. The programme primarily emphasised the reduction of carbohydrates (by 50 g per meal if BMI > 25 kg/m^2^) [57]. The incidence rate of diabetes in both groups was much higher than in other lifestyle modification programmes, which focused on overall calorie reduction and balanced meals [58]. In a systematic review and meta-analysis of observational studies, a long-term effect of the low-carbohydrate/ketogenic diet was associated with a higher risk of all-cause mortality, including cardiovascular disease (CVD) death [59], but reduced mortality when they were exchanged with plant-based carbohydrates [60]. In line with the case-control study on 225 newly diagnosed MASLD in Iranians, a high quality of carbohydrates, characterised by higher intakes of whole grain and low glycaemic index carbohydrates, was associated with a lower risk of MASLD [61]. These findings emphasised the quality of carbohydrates rather than their quantity.

### 4.2. Potential Mechanisms by Which Higher Dietary Fibre/CHO Ratio May Be Associated with Improved Cardiometabolic Parameters

Dietary fibre is an indigestible carbohydrate and that may be beneficial in reducing serum cholesterol, blood pressure [62,63], liver fat [64,65] and glucose excursion [66,67] by delaying the absorption of foods, and interaction with the gut microbiome [68,69,70]. These fibre-microbiome communications induce the production of short-chain fatty acids (SCFAs), essential vitamins, amino acids and neurotransmitters that are crucial for involving the gut–brain axis [71], immune response [72], and adipose tissue [73]. The anti-inflammatory role of SCFAs in the differentiation of beta cell function, stimulation of glucagon-like peptide-1 (GLP-1) and somatostatin hormone secretion. Moreover, some fibre-induced microbiota mimic the immunomodulatory activity, which improves insulin sensitivity [69]. These mechanisms regulate lipid profiles and beta-cell function [74]. Consistent with other studies [75,76], higher dietary fibre was correlated with lower body weight, blood pressure, and insulin resistance in our prediabetic cohort. Moreover, we also observed habitual soluble fibre in our study was correlated with other lipid profiles, including HDL and non-HDL, which are consistent results with soluble fibre supplementation [77]. However, the habitual consumption of dietary soluble fibre intake is still inadequate in the study cohort and much less than the dietary recommendation (≥7–13 g/d of soluble fibre) [78].

### 4.3. Associatios Between Protein/CHO and Fat/CHO Ratios with Lipid Profiles

Similar to the fibre/CHO ratio, we also observed that higher protein/CHO or fat/CHO ratios were correlated with lower triglycerides and higher HDL levels. However, these correlations disappeared after adjusting for age and gender. In a long-term prospective community-based Korean cohort with a mean of 7.7 years of follow-up, a higher protein/CHO ratio was associated with an increased risk of metabolic syndrome in men, not women [20]. In previous studies, a high-protein and low carbohydrate diet upregulates glucose production, increases glycogen turnover and stimulates gluconeogenesis, which may affect long-term cardiometabolic health [79,80]. In exchange for dietary fat, a low-carbohydrate, high-fat diet also increased LDL levels with an interventional effect of 1.82 mmol/L after 4 weeks of feeding trial in women who are healthy and of normal weight [81]. These findings underscore the potential influence of dietary exchanges for subjects at high risk of diabetes, the increased quality of carbohydrates, as measured by fibre/CHO ratio, rather than increased protein and fat in their diet to avoid cardiometabolic risk.

### 4.4. Limitations

Our study was limited by a modest sample size. However, we used detailed prospective food records, which are more time-consuming but provide more accurate information on daily dietary intake than the simple food frequency questionnaire [36,37]. We acknowledge limitations of 3-day dietary records where self-reported dietary intake could be underreported or misreported by subjects. Therefore, we verified the accuracy of self-reported diaries by additionally comparing with photo records by study dietitians/nutritionists. Our results indicated a weak-to-moderate correlation between the fibre/CHO ratio and lipid variables, which explained only a small portion of the variance in metabolic parameters. Genetic predisposition and other lifestyle factors may also contribute to cardiometabolic risk. Furthermore, since the observed association does not imply causation, our findings serve as a basis for generating hypotheses. We plan to perform future prospective longitudinal studies to evaluate the role of fibre/CHO ratios with insulin resistance as well as interventional studies to confirm our findings. Consequently, future interventional studies, including investigations of systematic inflammation, biomarkers, and gut microbiota, are necessary to confirm the impact of carbohydrate quality on metabolic outcomes related to prediabetes.

## 5. Conclusions

The fibre/CHO ratio could be an excellent indicator for accessing the adequate intake of quality carbohydrates necessary to improve blood pressure, lipid profiles, liver fat and insulin resistance. An increase in the fibre/CHO ratio was associated with lower cardiometabolic risk in people with prediabetes. Our study suggests that the quality of carbohydrates outweighs the quantity when it comes to increased fibre intake. For every 100 g of carbohydrates consumed, there should be more than 5.5 g of fibre derived from non-starchy vegetables and fruits. Considering this observation and supporting evidence, this straightforward nutritional index may be helpful in optimising nutritional interventions in people with prediabetes.

## Figures and Tables

**Table 1:** Baseline demographics stratified by gender. Abbreviation: BMI: body mass index, PG: plasma glucose, AUC: area-under-curve, HOMA: homeostasis model assessment, IR: insulin resistance, β: beta, S: sensitivity. Comparisons were made using Mann–Whitney or independent samples *t*-tests, with bold *p*-values of less than 0.05.

Variable	Total Cohort (*n* = 177)	Men (*n* = 72)	Women (*n* = 105)	*p*-Value
Age, years	60 (54–62)	60 (57–62)	60 (52–62)	0.501
Male, n (%)	72 (41)	NA	NA	NA
Statins, n (%)	66 (37.3)	33 (45.8)	33 (31.4)	0.059
Antihypertensive drugs, n (%)	76 (42.9)	38 (52.8)	38 (36.2)	**0.032**
Weight, kg	70.6 ± 12.9	78.1 ± 12.1	65.5 ± 10.8	**<0.0001**
Waist circumferences, cm	93.5 ± 9.8	96.5 ± 9.7	91.5 ± 9.4	**0.001**
Hip circumferences, cm	99.8 ± 7.5	100.3 ± 7.4	99.5 ± 7.7	0.508
BMI, kg/m^2^	26.7 ± 3.9	27.0 ± 3.7	26.5 ± 4.1	0.429
Systolic blood pressure, mmHg	133 ± 16.6	133 ± 16	133 ± 17	0.999
Diastolic blood pressure, mmHg	82.9 ± 10.4	86.3 ± 9.8	80.6 ± 10.1	**<0.0001**
Body fat, %	31.8 ± 8.7	25.1 ± 5.3	36.4 ± 7.4	**<0.0001**
Fasting plasma glucose, mmol/L	5.3 ± 0.5	5.4 ± 0.5	5.3 ± 0.5	0.646
1 h plasma glucose, mmol/L	10.9 ± 1.6	11.1 ± 1.8	10.8 ± 1.5	0.269
2 h plasma glucose, mmol/L	8.4 ± 1.4	8.2 ± 1.6	8.5 ± 1.2	0.106
AUC-PG mmol/L.min^−1^	18.5 ± 1.9	18.5 ± 2.0	18.6 ± 1.8	0.875
Fasting plasma CP, pmol/L	563 (434–742)	635 (478–796)	520 (352–712)	**0.008**
2 hr plasma CP, pmol/L	2955 (2295–3757)	3141 (2537–3809)	2774 (2270–3668)	0.130
HOMA2-IR	1.27 (0.94–1.67)	1.37 (1.07–1.78)	1.16 (0.80–1.54)	**0.009**
HOMA2-β (%)	99.9 (77.0–125.5)	111.8 (81.3–130.8)	93.2 (73.7–117.8)	0.057
HOMA2-S (%)	78.4 (59.0–104.1)	72.8 (56.3–93.1)	85.4 (63.9–118.7)	**0.015**
Lipid profiles				
Total cholesterol, mmol/L	4.9 ± 1.0	4.7 ± 1.1	5.1 ± 0.9	**0.026**
LDL, mmol/L	3.0 (2.3–3.5)	2.8 (2.1–3.5)	3.0 (2.4–3.6)	0.239
HDL, mmol/L	1.3 (1.1–1.6)	1.1 (1.0–1.3)	1.1 (1.3–1.7)	**<0.0001**
Triglycerides, mmol/L	1.2 (0.9–3.5)	1.3 (0.9–1.5)	1.1 (0.9–1.5)	0.139
Hepatic parameters				
Liver stiffness score (kPa)	4.4 (3.9–5.3)	4.7 (3.9–5.5)	4.4 (3.8–5.2)	0.316
CAP score (dB/m)	264 ± 54	267 ± 51	262 ± 56	0.645
Physical Activities				
Vigorous, MET-min/week	0 (0–0)	0 (0–0)	0 (0–240)	0.369
Moderate, MET-min/week	0 (0–480)	0 (0–720)	120 (0–480)	0.980
Light, MET-min/week	693 (330–1386)	693 (297–1386)	693 (347–1386)	0.428
Total physical activity MET-min/week	1166 (484–2243)	1188 (594–2772)	1208 (495–2316)	0.815
Sedentary, min/day	300 (180–480)	300 (180–480)	300 (180–420)	0.429

Abbreviations: BMI: body mass index, LDL: low-density lipoprotein, HDL: high-density lipoprotein, HOMA2-IR: homeostatic model assessment for insulin resistance, HOMA2-B: homeostatic model assessment for beta cell function, HOMA2-S: homeostatic model assessment for insulin sensitivity, CP: C-peptide, CAP: controlled attenuation parameter, MET: metabolic equivalent of task. NA for not applicable. Normal ranges for fasting glucose levels are less than 5.6 mmol/L, 2 h glucose < 7.8 mmol/L, total cholesterol < 5.2 mmol/L, triglycerides < 1.7 mmol/L, HDL > 1.0 mmol/L, and LDL < 2.6 mmol/L.

**Table 2:** Macronutrients, carbohydrates ratio and minerals intake. Comparisons were made using Mann–Whitney or independent samples *t*-tests, with bold *p*-values of less than 0.05.

Variable	Total Cohort (*n* = 177)	Men (*n* = 72)	Women (*n* = 105)	*p*-Value
Dietary information				
Energy, kcal/day	1885 (1553–2182)	2110 (1809–2515)	1757 (1454–2046)	**<0.0001**
Carbohydrates, g/day	201 (165–248)	232 (183–274)	190 (155–234)	**<0.0001**
Protein, g/day	87 (72–102)	98 (85–115)	81 (64–90)	**<0.0001**
Protein/CHO	0.42 (0.34–0.52)	0.44 (0.36–0.54)	0.42 (0.33–0.51)	0.176
Fat, g/day	80 ± 25	89.6 ± 25	73.7 ± 23	**<0.0001**
Saturated fat, g/day	19.4 (15.5–24.4)	21.5 (18.1–27.0)	12.3 (13.0–21.7)	**<0.0001**
Fat/CHO	0.37 (0.30–0.47)	0.38 (0.30–0.47)	0.36 (0.29–0.65)	0.678
Fibre, g/day	11 (8–15)	10 (8–15)	12 (8–16)	0.232
Soluble fibre, g/day	0.70 (0.36–1.26)	0.59 (0.33–1.29)	0.77 (0.42–1.22)	0.232
Fibre/CHO	0.05 (0.04–0.08)	0.05 (0.03–0.07)	0.06 (0.04–0.08)	**<0.0001**
Total sugar, g/day	41 (29–58)	47 (31–60)	39 (27–56)	0.080
Minerals				
Potassium, mg	2291 (1726–2959)	2331 (1849–3014)	2202 (1646–2955)	0.092
Sodium, mg	3743 (3104–4528)	3975 (3524–4999)	3620 (2867–4248)	**0.008**
Sodium/potassium ratio	1.72 (1.18–2.17)	1.76 (1.27–2.17)	1.68 (1.17–2.19)	0.836

**Table 3:** Multivariate analysis of associations between fibre/CHO ratio and blood pressure, plasma triglycerides and HOMA2-IR.

Dependent Variable (fibre/CHO)	Standardised Beta Coefficient	95% CI	Adjusted R^2^	*p* Value
Systolic blood pressure (mmHg)				
Base model	−0.232	[−188.74 to −41.15]	0.092	**0.002**
Model 1	−0.245	[−200.24 to −41.74]	0.139	**0.003**
Model 2	−0.281	[−227.5 to −56.35]	0.157	**0.001**
Model 3	−0.277	[−236.32 to −43.48]	0.151	**0.005**
Model 4	−0.281	[−231.94 to −51.83]	0.145	**0.002**
Triglycerides (mmol/L)				
Base model	−0.198	[−9.908 to −1.299]	0.054	**0.011**
Model 1	−0.176	[−9.612 to −0.356]	0.103	**0.035**
Model 2	−0.190	[−10.929 to −0.531]	0.119	**0.031**
Model 3	−0.270	[−13.889 to −2.361]	0.134	**0.006**
Model 4	−0.195	[−11.332 to −0.410]	0.110	**0.035**
Fatty liver, CAP (dB/m)				
Base model	−0.249	[−696.8 to −64.6]	0.047	**0.019**
Model 1	−0.271	[−782.0 to −45.9]	0.073	**0.028**
Model 2	−0.252	[−747.3 to −21.2]	0.108	**0.038**
Model 3	−0.308	[−866.9 to −74.3]	0.109	**0.020**
Model 4	−0.259	[−787.3 to −5.02]	0.097	**0.047**
Insulin resistance, HOMA2-IR				
Base model	−0.272	[−7.286 to −2.225]	0.150	**<0.0001**
Model 1	−0.276	[−7.581 to −2.044]	0.160	**0.001**
Model 2	−0.263	[−7.866 to −1.726]	0.170	**0.002**
Model 3	−0.252	[−8.024 to −1.148]	0.165	**0.009**
Model 4	−0.237	[−7.529 to −1.098]	0.165	**0.009**

Plasma systolic blood pressure, triglycerides, CAP, and HOMA2-IR were included as dependent variables, with the fibre/CHO ratio as an independent variable. Base model: adjusted for age, gender, lipid-lowering drugs, and antihypertensive drugs; Model 1 = base model + total calorie intake, protein, fat, and total sugar; Model 2 = Model 1 + physical activities (total physical activity and sedentary); Model 3 = Model 2 + sodium/potassium ratio; Model 4 = Model 2 + education status (college, high secondary, middle secondary, primary and no formal education) and alcohol consumption (ex-drinker, social drinker, current drinker, and never). Bold was *p*-value < 0.05. Abbreviations: Controlled attenuation parameter (CAP), homeostasis model assessment for insulin resistance (HOMA2-IR).

**Table 4:** Binary logistic regression model for reducing hypertriglyceridaemia (>1.7 mmol/L) by the fibre/CHO ratio quartiles.

Triglycerides (>1.7 mmol/L)	Unadjusted Model OR (95% CI), *p*-Value	Model 1 OR (95% CI), *p*-Value	Model 2 OR (95% CI), *p*-Value
1st quartile (<0.038 fibre/CHO ratio)	Ref	Ref	Ref
2nd quartile (0.038–0.055) fibre/CHO ratio)	0.123 (0.014–1.046), *p* = 0.598	0.139 (0.016–1.218), *p* = 0.075	0.145 (0.016–1.273), *p* = 0.081
3rd quartile (0.055–0.076 fibre/CHO ratio)	0.079 (0.010–0.648), ***p* = 0.018**	0.081 (0.010–0.667), ***p* = 0.019**	0.078 (0.009–0.645), ***p* = 0.018**
4th quartile (>0.076 fibre/CHO ratio)	0.079 (0.010–0.648), ***p* = 0.018**	0.080 (0.010–0.659), ***p* = 0.019**	0.072 (0.009–0.603), ***p* = 0.015**

Hypertriglyceridaemia (>1.7 mmol/L) was included as the dependent variable, with the fibre/CHO ratio as the independent variable. Each quartile represents 25% of the data. Fibre/CHO ratio less than 0.038 as a reference group. Bold was *p*-value < 0.05. Model 1: adjusted for age and gender. Model 2: Model 1 plus adjusted for lipid-lowering and antihypertensive drugs.

## Data Availability

The data from this study can be requested from the corresponding author. These data are not publicly available due to privacy restrictions.

## Supplementary Materials

The following supporting information can be downloaded at https://www.mdpi.com/article/10.3390/nu17071123/s1.
