# Supplementary material for: Carbohydrate Quality Is Independently Associated with Cardiometabolic Risk in Chinese Individuals with Impaired Glucose Tolerance
Source: Nutrients. 2025 Mar 24;17(7):1123. doi: 10.3390/nu17071123 (PMC11990533; doi:10.3390/nu17071123)
Supplement: Supplementary file 1 [file nutrients-17-01123-s001.zip › nutrients-3527940-supplementary.pdf]

Diet and activity record sheet

Name:______________

Study number: ________________

Study Stage: _______________

Diet and activity record sheet guidelines

1. Please fill in the diet and activity records from ____________to___________.
2. Please do not change your daily eating/activity habits by filling in this diet and activity record.
3. If possible, please write down the weight/portion size of the food. If it is a prepackaged food, you can write down the weight on the label. If it is hard to do, you can keep a low portion size in a simple way on a daily basis, such as 2 teaspoons of sugar, 2 tablespoons of vegetables, a bowl of rice, etc.
4. Please fill in the cooking method, such as frying, steaming, frying or boiling.
5. Please make a brief note of your daily activities and exercise, and sleep time, such as walking for 30 minutes, Tai Chi for 45 minutes; Sleep Time: 10:00pm – 8:00am.

Diet and Activity Record (Sample)

Date： 4.1.2019 (Friday)

Time	Venue	Food/Drink intake	Amount
7:10 am	home	Plain Bread (with crust)	1 slice
		Pan fried egg	1 egg
		Full cream milk	1glass
10:30 am	office	Cheese cracker	1pack
		Marshmallow	2 pieces
		water	1glass
1:30 pm	restaurant	Streamed rice (with octopus and, chicken and chicken feet)	1 lunchbox
		Chicken feet	2 pieces
		Octopus and chicken	4 tablespoons
		Iced milk tea (less sugar)	1 glass
4:30 pm	office	Pork jerky	2 small pieces
		water	1 glass
8:00 pm	home	rice	Half bowl
		Pan grilled pork chop	2 pieces
		Fried choi-sum	4pieces
		Hairy melon and bean curd stew	4 pieces each
		Winter melon and lean pork soup	1 bowl
		Lean pork (in soup)	4 pieces
9:00 pm	home	Pear (peeled)	Half pear
10:30 pm	home	Sago dessert	Half bowl

Water intake for whole day：___2_____ glass/ ml (ml circled)   supplement (vitamin/ calcium) (if any)：Calcium 1tab__

Cooking oil used：____corn oil______   seasoning (salt, soy sauce): ☐light ☑medium ☐heavy

Daily activity and sports: _walk 15 mins___   wake up time: _8:30 am_____   bedtime: _11:30pm_____

Remarks**:** ____

Diet and Activity Record (1)

Please record all foods eaten, including breakfast, lunch, dinner, soup, drinks, snacks and evening meals.

Please use bowls, cups, tablespoons, teaspoons, large/small pieces, large/small pieces, or sizes as the unit of record.

Date：__________________ (________)

				**(office use)**	
**Time**	**Venue**	**Food/ Drink**	**Amount**	編號	三天份量

Water intake for whole day：____________glass/ ml   supplement (if any)：______________________

Cooking oil used：_______________   seasoning (salt, soy sauce): ☐light ☐medium ☐heavy

Daily activity and sports:______________   wake up time: ______________

bedtime: _______________________

Diet and Activity Record (2)

Please record all foods eaten, including breakfast, lunch, dinner, soup, drinks, snacks and evening meals.

Please use bowls, cups, tablespoons, teaspoons, large/small pieces, large/small pieces, or sizes as the unit of record.

Date：__________________ (________)

				**(office use)**	
**Time**	**Venue**	**Food/ Drink**	**Amount**	編號	三天份量

Water intake for whole day：____________glass/ ml supplement (if any)：______________________

Cooking oil used：_______________ seasoning (salt, soysauce): ☐light ☐medium ☐heavy

Daily activity and sports:______________ wake up time: ______________ bedtime:__________________

Diet and Activity Record (2)

Please record all foods eaten, including breakfast, lunch, dinner, soup, drinks, snacks and evening meals.

Please use bowls, cups, tablespoons, teaspoons, large/small pieces, large/small pieces, or sizes as the unit of record.

Date：___________________ (________)

				**(office use)**	
**Time**	**Venue**	**Food/ Drink**	**Amount**	編號	三天份量

Water intake for whole day：____________glass/ ml supplement (if any)：______________________

Cooking oil used：_______________ seasoning (salt, soysauce): ☐light ☐medium ☐heavy

Daily activity and sports:______________ wake up time: ______________ bedtime:__________________

Diet Record Date (   )

MealTime Veneu Food(full description)

_______ ____ ____________________

_______ ____ ____________________

_______ ____ ____________________

_______ ____ ____________________

_______ ____ ____________________

_______ ____ ____________________

_______ ____ ____________________

_______ ____ ____________________

_______ ____ ____________________

_______ ____ ____________________

Activity ____________________

____

Bedtime and others ____________________

Diet Record Date (   )

MealTime Veneu Food(full description)

_______ ____ ____________________

_______ ____ ____________________

_______ ____ ____________________

_______ ____ ____________________

_______ ____ ____________________

_______ ____ ____________________

_______ ____ ____________________

_______ ____ ____________________

_______ ____ ____________________

_______ ____ ____________________

Activity ____________________

____

Bedtime and others ____________________

Diet Record Date (   )

MealTime Veneu Food(full description)

_______ ____ ____________________

_______ ____ ____________________

_______ ____ ____________________

_______ ____ ____________________

_______ ____ ____________________

_______ ____ ____________________

_______ ____ ____________________

_______ ____ ____________________

_______ ____ ____________________

_______ ____ ____________________

Activity ____________________

____

Bedtime and others ____________________

Diet Record Date (   )

MealTime Veneu Food(full description)

_______ ____ ____________________

_______ ____ ____________________

_______ ____ ____________________

_______ ____ ____________________

_______ ____ ____________________

_______ ____ ____________________

_______ ____ ____________________

_______ ____ ____________________

_______ ____ ____________________

_______ ____ ____________________

Activity ____________________

____

Bedtime and others ____________________

Diet Record Date (   )

MealTime Veneu Food(full description)

_______ ____ ____________________

Diet Record Date (   )

MealTime Veneu Food(full description)

_______ ____ ____________________

Diet Record Date (   )

MealTime Veneu Food(full description)

_______ ____ ____________________

Diet Record Date (   )

MealTime Veneu Food(full description)

_______ ____ ____________________

________	____	____________________	________	____	____________________	________	____	____________________	________	____	____________________
________	____	____________________	________	____	____________________	________	____	____________________	________	____	____________________
________	____	____________________	________	____	____________________	________	____	____________________	________	____	____________________
________	____	____________________	________	____	____________________	________	____	____________________	________	____	____________________
________	____	____________________	________	____	____________________	________	____	____________________	________	____	____________________
________	____	____________________	________	____	____________________	________	____	____________________	________	____	____________________
________	____	____________________	________	____	____________________	________	____	____________________	________	____	____________________
________	____	____________________	________	____	____________________	________	____	____________________	________	____	____________________
________	____	____________________	________	____	____________________	________	____	____________________	________	____	____________________
Activity		____________________	Activity		____________________	Activity		____________________	Activity		____________________
________________________________			________________________________			________________________________			________________________________		
Bedtime and others		____________________	Bedtime and others		____________________	Bedtime and others		____________________	Bedtime and others		____________________

IPAQ Physical activity questionnaire

This questionnaire explores how people participate in physical activity on a daily basis, and the questionnaire is about the time you have spent participating in physical activity in the past seven days. Even if you are not an active person, please answer each question. Think carefully about your daily activities including work, chores, gardening, travel to and from various locations, and recreational, sporting or sporting activities in your free time.

Consider the strenuous physical activity you've had in the last seven days, which is when it takes a lot of energy and makes your breathing harder than usual. Try just those physical activities that you do for at least ten minutes at a time.

1. How many days have you spent in strenuous physical activity in the last seven days, such as lifting heavy objects, digging the ground, dancing health or speed cycling?
   _________days per week ☐ No strenuous physical activity, please go to question 3
2. How much time do you usually spend on strenuous physical activity on one of the last seven days?
   __________hours__________mins per day ☐ Unknown

Consider the moderate physical activity you've had in the last seven days, which requires moderate physical exertion and makes your breathing harder than usual. Try just those physical activities that you do for at least ten minutes at a time.

3. How many days have you spent in moderate physical activity in the last seven days, such as lifting light objects, speeding bikes or tennis doubles? Walking is not included.
   _________days per week ☐ No moderate physical activity, please go to question 5

4. How much time do you usually spend on moderate physical activity on one of the last seven days?

__________hours__________mins per day ☐ Unknown

Think of the time you have spent walking in the last seven days, including at work or at home, on foot by transport, and on foot for recreational, athletic or leisure activities

5.How many days have you spent in walking (more than 10 minutes) in the the last seven days?

_______________days per week ☐ No walking, please go to question 7

6. How much time do you usually spend on walking on one of the last seven days?

__________hours__________mins per day   Unknown

7. The last question is about the time you have spent sitting in the last seven days (not including Saturdays and Sundays), which includes time at work, at home, in further study, and leisure, which can include reading at your desk, visiting friends, reading, or sitting or lying down watching TV.

   __________hours__________mins per day   ☐ Unknown
